# Small-angle X-ray scattering of engineered antigen-binding fragments: the case of glycosylated Fab from the Mannitou IgM antibody

**DOI:** 10.1107/S2053230X24012159

**Published:** 2025-01-01

**Authors:** Shubham Semwal, Maria Karamolegkou, Stéphanie Flament, Nessim Raouraoua, Kenneth Verstraete, Aurélien Thureau, Frank Wien, Fabrice Bray, Savvas N. Savvides, Julie Bouckaert

**Affiliations:** a Unité de Glycobiologie Structurale et Fonctionnelle (UGSF), UMR 8576 CNRS and University of Lille Villeneuve d’Ascq France; b VIB–UGent Center for Inflammation Research, Ghent, Belgium; c Unit for Structural Biology, Department of Biochemistry and Microbiology Ghent University Ghent Belgium; d University of Lille, CNRS, UAR 3290–MSAP–Miniaturisation pour la Synthèse, l’Analyse et la Protéomique, 59000Lille, France; e Synchrotron SOLEIL L’Orme des Merisiers, Saint-Aubin BP48 91192Gif-sur-Yvette France; University of York, United Kingdom

**Keywords:** Fabs, SEC-SAXS, SEC-MALS, SRCD, glycosylation

## Abstract

Monoclonal antibodies recognizing nonprotein antigens remain largely underrepresented in our understanding of the molecular repertoire of innate and adaptive immunity. One such antibody is Mannitou, a murine IgM that recognizes paucimannosidic glycans. In this work, we report the production and purification of the recombinant antigen-binding fragment (Fab) of Mannitou IgM (Mannitou Fab) and employ a combination of biochemical and biophysical approaches to obtain its initial structural characterization. To this end, recombinant Mannitou Fab comprising the light chain (VL-CL) and heavy chain (VH-Cμ1) was produced in HEK293 FreeStyle cells and purified by cobalt-affinity chromatography followed by size-exclusion chromatography (SEC), which revealed two distinct oligomeric states consistent with a predominant monomeric form and a minor dimeric form. We employed SEC inline with multi-angle light scattering (SEC-MALS) and SEC coupled to small-angle X-ray scattering (SEC-SAXS) to establish that Mannitou Fab indeed adopts monomeric and dimeric forms in solution. Interestingly, Mannitou Fab is N-glycosylated at Asn164 of the heavy chain via HexNAc(5)Hex(6)Fuc(1–3) as revealed by mass spectrometry. We leveraged this information in conjunction with predicted structures of Mannitou Fab to facilitate the interpretation and modelling of SAXS data, leading to a plausible model for glycosylated Mannitou Fab. Analysis of the two chromatographically isolatable forms of Mannitou Fab using synchrotron-radiation circular dichroism revealed that the heat-denaturated Mannitou Fab monomer shares similar secondary-structural elements with the Mannitou Fab dimer, indicating that the latter may be misfolded. Collectively, the findings of this study will set the stage for future structural studies of Mannitou Fab and contribute to our understanding of possible side products due to misfolding during the production of recombinant Fabs, highlighting the importance of glycosylation in obtaining stable and monodisperse monomeric forms of recombinant Fabs.

## Abstract 2

This study provides a biochemical and biophysical analysis of Mannitou Fab, highlighting the intricate balance between stability, flexibility and function in engineered antibody fragments. The results underscore the critical role of glycosylation in modulating the oligomerization behaviour of Fab and preventing inappropriate intermolecular interactions, paving the way for more effective and reliable therapeutic antibodies in the future.

## 1. Introduction

The field of antibody engineering has experienced remarkable advancements, particularly with the emergence of monoclonal antibodies that have transformed therapeutic strategies for various diseases, including cancers and autoimmune disorders (Freise & Wu, 2015 ▸; Kaveri *et al.*, 2012 ▸). The ability to design and optimize antibody fragments, such as the fragment antigen-binding (Fab) regions, has become a focal point in this domain due to their high specificity and reduced immunogenicity (Ruffolo *et al.*, 2023 ▸). These fragments retain the essential antigen-binding domains while lacking the constant regions that typically activate immune responses, making them particularly suitable for targeting diseases where immune activation could be detrimental (Sloan *et al.*, 2015 ▸). However, engineering these antibody fragments presents several challenges, notably in optimizing the binding affinity, stability and expression levels (Wang *et al.*, 2022 ▸). The complexity of antibody structure, influenced by factors such as glycosylation and other post-translational modifications, plays a crucial role in determining the efficacy and stability of Fab-based therapeutics (Righi *et al.*, 2023 ▸).

The comparative study of immunoglobulin M (IgM) versus immunoglobulin G (IgG) reveals significant differences in their structural characteristics and binding mechanisms, which have functional implications in immune responses. IgM is typically a pentameric, and sometimes a hexameric, structure, which confers unique properties compared with the monomeric form of IgG (Sharp *et al.*, 2019 ▸). Structural analysis of IgM using cryo-electron microscopy (cryo-EM) has demonstrated a flexible hinge motion that allows a distinct binding orientation of its Fab arms (Chen *et al.*, 2022 ▸). This facilitates simultaneous engagement with up to 12 antigens, in contrast to IgG where typically only one Fab arm engages with an antigen while the other remains unbound, leading to a less effective binding profile in certain contexts (Sharp *et al.*, 2019 ▸). Furthermore, the binding dynamics of IgM and IgG are influenced by their respective Fab fragments. Studies have shown that IgM exhibits a higher affinity for late apoptotic cells compared with IgG, which is attributed to their ability to recognize specific antigens that are exposed during apoptosis (Mayumi *et al.*, 1995 ▸; Hiramoto *et al.*, 2018 ▸). This property is crucial for the clearance of apoptotic cells and suggests that IgM plays a vital role in maintaining immune homeostasis, a function that IgG does not fulfil as effectively (Goldberg & Ackerman, 2020 ▸). For example, the majority of rheumatoid factors are IgM autoantibodies that occur in rheumatoid arthritis as well as in normal immune responses. IgM rheumatoid factors and IgGs interact via their Fc portion in immune complexes with the antigens. The soluble ‘decoy’ Fc receptor FcγRIIa specifically targets the Fc portion of IgM rheumatoid factor and disrupts Fc–Fc interactions in immune complexes, thereby decreasing the inflammatory response (Wines *et al.*, 2003 ▸). The engineering of IgM antibodies is challenging due to their structural complexity and stability issues compared with IgG (Shrestha, 2020 ▸). However, the unique properties of IgM, such as its ability to activate complement more effectively than IgG (John *et al.*, 2024 ▸), highlight its potential in therapeutic contexts where rapid immune responses are required.

Recent studies have emphasized the importance of understanding IgM modifications to enhance the therapeutic potential of antibody fragments. For instance, this work provides insights into the structural and functional characterization of a Fab fragment from IgM Mannitou (Bajt *et al.*, 1990 ▸; Zipser *et al.*, 2012 ▸; Robakiewicz *et al.*, 2021 ▸), highlighting the significance of Fab-fragment glycosylation. A range of biophysical techniques, including size-exclusion chromatography (SEC), multi-angle light scattering (MALS), small-angle X-ray scattering (SAXS) and synchrotron-radiation circular dichroism (SRCD), have been employed to assess the structural integrity and oligomerization tendencies of antibody fragments. Mass-spectrometric techniques for protein and glycopeptide analyses, to identify the nature of the glycosylation, together with *in silico* structure prediction (*AlphaFold*3) and *in silico* glycosylation (*GlycoSHIELD*) have helped to improve the Mannitou Fab model that fits the SAXS data. The comprehensive approach taken in the study of antibody fragments not only aims to clarify their monomeric and oligomeric states but also seeks to identify the impact of glycosylation on their structural properties, thereby contributing to the broader understanding of recombinant antibody engineering.

## 2. Materials and methods

### 2.1. Design of the antigen-binding fragment of Mannitou IgM

To design the antigen-binding fragment (Fab) of Mannitou IgM (Robakiewicz *et al.*, 2021 ▸), two constructs of complementary DNA (cDNA) segments encoding the N-terminal variable domain and the first constant domain of the light and the heavy chain, respectively, were codon-optimized for expression in human cells, synthesized (GenScript) and subcloned into two separate pHLSec expression vectors (Aricescu *et al.*, 2006 ▸). The leader sequences were not included in the constructs but were replaced by the RPTμ secretion signal. The heavy chain was tagged with a six-histidine (His_6_) tag at its C-terminus to allow affinity purification and detection of the heavy chain on a Western blot using anti-His antibody.

### 2.2. Production and purification of antigen-binding fragment (Fab)

For Mannitou Fab expression, HEK293 FreeStyle suspension cells (Thermo Fisher Scientific) were cultured at 37°C in FreeStyle 293 Expression medium (Gibco). Upon reaching >95% viability, the cell density was adjusted to 0.7 × 10^6^ cells ml^−1^ to reach approximately 1 × 10^6^ cells ml^−1^ on the day of transfection. Transfection was carried out using linear polyethylenimine (PEI) as the transfection agent, with a 2:1 ratio of DNA over PEI, for a total mass of 1 µg of DNA per millilitre of cells. Equal amounts of light-chain (VL-CL) and heavy-chain (VH-Cμ1) DNA were added. The DNA–PEI complex was allowed to form over 20 min at room temperature in Opti-MEM Reduced Serum Medium (Gibco, catalogue No. 31985062) before being transfected in the cells. Post-transfection, 1% penicillin–streptomycin was added to the culture. The following day, Ex-Cell serum-free medium was added to the culture, making up 20% of the total transfection volume. The produced Fab was harvested 3–4 days post-transfection by recuperating the culture medium and removing the cells by centrifugation at 600*g* for 15 min. The supernatant containing the Fab protein was purified from the conditioned medium using two different columns: immobilized cobalt-affinity chromatography followed by size-exclusion chromatography (SEC). Cobalt was selected over nickel to minimize nonspecific binding and co-purification of contaminants, which is a common issue with nickel-based resins. The supernatant was filtered through a 0.22 µm PES filter (Millipore) and passed through the cobalt-affinity column pre-equilibrated in phosphate buffer pH 7.4 complemented with 500 m*M* NaCl. Upon washing and return to the baseline, elution was achieved using a gradient of 500 m*M* imidazole. Next, the recombinant Mannitou Fab fractions were dialysed against 20 m*M* HEPES pH 7.4, 300 m*M* NaCl. Dialysis was necessary to prevent aggregation of the His-tagged protein during the concentration step, which required a reduction in the sample volume (a maximum of 0.5 ml) loaded onto the gel-filtration column. The latter was performed in the same buffer as the dialysis buffer on a Superose 6 Increase 10/300 GL column (Cytiva). Mannitou Fab production information is summarized in Table 1 ▸.

### 2.3. SEC-MALS

The Mannitou Fab samples thus separated on a Superose 6 Increase 10/300 GL column (Cytiva) were further analysed by SEC-MALS. Prior to this native molecular-weight analysis, peaks A and B were visualized using a 4–16% stain-free gradient gel (Bio-Rad) under denaturing conditions, either nonreduced or reduced using 100 m*M* tris(2-carboxyethyl)phosphine (TCEP). This SDS–PAGE gel was imaged using UV fluorescence Subsequently, the proteins were transferred from the gel onto nitrocellulose and revealed using His_6_ Tag Antibody Dylight 800 Conjugated (Rockland Immunochemicals) to identify the presence of the heavy chain.

Both Superdex 200 Increase 10/300 GL and Superose 6 Increase 10/300 GL columns were utilized and were connected to a Shimadzu UV detector, a mini-DAWN TREOS multi-angle laser-light scattering (MALS) instrument (Wyatt) and an Optilab T-rEX refractometer (Wyatt). A d*n*/d*c* (refractive-index increment) value of 0.185 ml g^−1^ was used to determine the molecular mass of the protein complex using the *ASTRA*6 software (Wyatt). To estimate the content of non-UV-absorbing post-translational modifications, a d*n*/d*c* of 0.15 ml g^−1^ was applied (Hastie *et al.*, 2021 ▸). Bovine serum albumin (BSA) was injected prior to the Fab samples, under identical conditions, to correct the band broadening and set the baseline parameters. The gel filtration was performed at a flow rate of 0.75 ml min^−1^ in 20 m*M* HEPES pH 7.4, 300 m*M* NaCl at ambient temperature.

### 2.4. SEC-SAXS data collection and processing

Size-exclusion chromatography coupled with small-angle X-ray scattering (SEC-SAXS) was performed using a Bio SEC-3 300 column (Agilent; 300 Å pore size, 4.6 × 300 mm) connected to an HPLC system at the SWING beamline at the SOLEIL synchrotron. The mobile phase consisted of 20 m*M* HEPES, 300 m*M* NaCl pH 7.5, which was filtered and degassed using 0.22 µm filters before use. The Bio SEC-3 300 column was pre-equilibrated with at least two column volumes of the running buffer at a flow rate of 0.3 ml min^−1^ to establish a stable baseline before sample injection. Mannitou Fab purified by cobalt-affinity chromatography was dialysed, as described in Section 2.2, before being concentrated to 9 mg ml^−1^. A 70 µl volume of the protein sample was injected into the column, and SEC was carried out at a flow rate of 0.3 ml min^−1^. The eluent was directly passed into the SAXS flow cell for continuous data collection, maintaining the integrity of the SEC-SAXS setup without fraction collection. SAXS data were recorded in real time as the protein sample eluted from the column, with an exposure time of 1 s per frame. The intensity was calibrated to absolute units using the scattering of pure water. Initial data processing was performed using *FOXTROT* for real-time visualization and primary reduction of the scattering data (Thureau *et al.*, 2021 ▸). At the same time, further refinement and analysis were performed using *BioXTas RAW* (Hopkins *et al.*, 2017 ▸), including the evolving factor analysis (EFA) deconvolution algorithm, along with the *ATSAS* software suite and its constituent programs *PRIMUS*, *GNOM* and *DAMMIF* (Manalastas-Cantos *et al.*, 2021 ▸). Buffer scattering profiles were subtracted from each protein scattering profile, and parameters such as the radius of gyration (*R*_g_), maximum particle dimension (*D*_max_) and pairwise distance distribution functions [*P*(*r*)] were calculated.

### 2.5. Mannitou Fab model generation

A model of Mannitou Fab was generated using *AlphaFold*3 (Abramson *et al.*, 2024 ▸), and the best prediction (ranked_0.pdb) was selected based on the highest confidence (predicted local distance difference test; pLDDT) and the lowest predicted aligned error (PAE; Jumper *et al.*, 2021 ▸). *AlphaFold*3 was initially used to model the Mannitou Fab structure, but its limitation of handling only eight monosaccharide residues made it unsuitable for modelling the full glycan of 11–14 residues. To address this, *GlycoSHIELD* (Tsai *et al.*, 2024 ▸) was employed to incorporate the complete glycan structure onto the *AlphaFold*3-predicted backbone, ensuring alignment with experimental mass-spectrometric (MS) data and SAXS constraints. Using a reduction-based molecular-dynamics analysis, *GlycoSHIELD* generated an ensemble of 30 glycan conformers on Mannitou Fab. In a final refinement, individual glycan conformers were extracted from the *GlycoSHIELD* multi-state model using *PyMOL* (version 2.4.0; DeLano, 2005 ▸) to fit the SAXS profiles using the *FOXS* program (Schneidman-Duhovny *et al.*, 2013 ▸). The residuals between the theoretical scattering curve generated from the model and the experimental scattering curve were represented in the χ^2^ parameter.

### 2.6. SAXS-based (ensemble) modelling

Molecular weights were calculated based on Guinier analysis of the scattering curve using *FoXS* (Schneidman-Duhovny *et al.*, 2010 ▸). The experimental scattering profiles were compared with theoretical profiles generated from atomic models using *CRYSOL* (Svergun *et al.*, 1995 ▸). SAXS-based ensemble modelling was performed using *BilboMD* (Pelikan *et al.*, 2009 ▸) and *MultiFoXS* (Schneidman-Duhovny *et al.*, 2016 ▸), generating 100 conformations per *R*_g_, with minimal and maximal *R*_g_ values set to 7% and 50% of the experimentally determined *R*_g_, respectively. *FoXS* was used to evaluate the fits of the models to the experimental data, while *BilboMD* explored conformational flexibility, focusing on dynamic regions. The overall fit between the models and the experimental data was assessed by calculating the χ^2^ value. Together, these tools ensured accurate and robust structural analysis.

### 2.7. MALDI-TOF and LC-MS-MS glycoproteomics

The protein identity and glycosylation status of Mannitou Fab samples were analysed using matrix-assisted laser desorption ionization–time of flight (MALDI-TOF) mass spectrometry and liquid chromatography with tandem mass spectrometry (LC-MS-MS).

For MALDI-TOF experiments, the matrix α-cyano-4-hydroxycinnamic acid (HCCA) was dissolved at a concentration of 10 mg ml^−1^ in 70% acetonitrile, 0.1% formic acid. 0.5 µl of the matrix was mixed with 0.5 µl of sample and deposited onto the MALDI plate. The calibration of the linear MALDI TOF-TOF 4800 (ABSciex) instrument in ion-positive mode was first verified using standard bovine serum albumin (BSA; 1 mg ml^−1^) in the same mid-mass range (20 000–90 000 *m*/*z*) in order to be able to observe single-, double- and triple-charged BSA. Deposited Mannitou Fab samples were diluted to concentrations of less than 1 mg ml^−1^ to avoid aggregation.

Mannitou Fab was prepared for glycoproteomics analysis using LC-MS-MS by trypsin digestion of 50 µg of the sample and following the eFASP method (Erde *et al.*, 2014 ▸). 1 µg of peptide material was injected for separation on a nanoHPLC U 3000 (Thermo Scientific) on a C18 column (75 µm, 50 cm C18) equipped with a pre-column (300 µm, 5 mm) at a flow rate of 250 nl min^−1^ and 45°C, using a 140 min gradient. The mass of the thus separated peptides was analysed using nanoelectrospray on an Orbitrap Q-Exactive plus (Thermo Scientific), with an MS resolution of 70 000 and an MS/MS resolution of 35 000. The *m*/*z* data on the peptides/glyco­peptides were analysed using *Byonic* (Bern *et al.*, 2012 ▸), with a filter for a false-discovery rate (FDR) of 1%.

### 2.8. Synchrotron-radiation circular dichroism (SRCD)

CD spectra were acquired using SRCD on the DISCO beamline of the SOLEIL synchrotron, Saint-Aubin, France (Turbant *et al.*, 2024 ▸). Samples of 5 µl of monomeric (peak A) and dimeric (peak B) Mannitou Fab, at different concentrations between 12 and 4 mg ml^−1^, were placed between two CaF_2_ cover slips, ensuring a path length of 52.5 µm. The beam minimized radiation damage with a size of 4 × 4 mm and a photon flux of 2 × 10^10^ photons s^−1^ nm^−1^ in the 270–170 nm range. SRCD spectra were collected over three consecutive accumulations and averaged. Buffer baselines were recorded and subtracted from the sample spectra, adjusting for residue concentration. The data were processed using *CDToolX* (Miles & Wallace, 2018 ▸) and the spectra were analysed for secondary-structure elements using *BeStSel* (Micsonai *et al.*, 2021 ▸).

## 3. Results and discussion

### 3.1. In Mannitou Fab production, higher-order oligomers are formed

**Figure 1:**
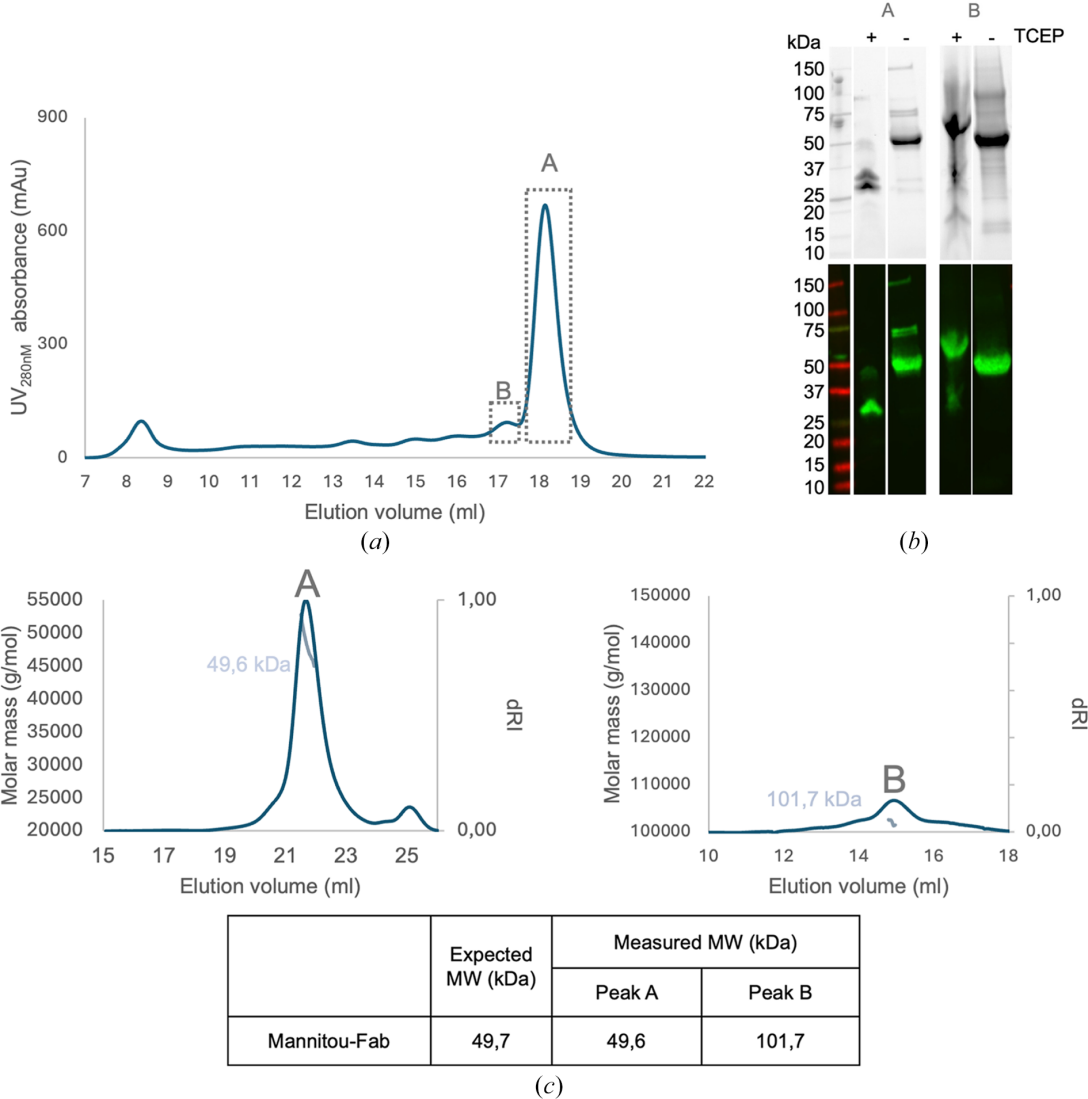
SEC-MALS analysis of Mannitou Fab. (*a*) Size-exclusion chromatography profile of Mannitou Fab on a Superose 6 Increase 10/300 column upon cobalt-affinity purification. The dashed boxes indicate the portions of the eluted protein (peaks A and B) used for further analyses. (*b*) SDS–PAGE gel of peaks A and B and their Western blot analysis using an anti-His antibody. (*c*) SEC-MALS analysis of peaks A and B of the Mannitou Fab produced in HEK293 FreeStyle cells. These analyses were performed using a Superdex 200 Increase column (peak A) and a Superose 6 Increase column (peak B). The inset table shows the theoretical molecular weight and the molecular weights based on the protein content as determined by SEC-MALS analysis.

The SEC profile of Mannitou Fab produced in HEK293 FreeStyle cells showed an abundant peak (labelled A) eluting at ∼18 ml from the Superose 6 Increase 10/300 GL column. In addition, several earlier-eluting peaks suggested the presence of higher-order molecular-weight species, of which we selected the peak (labelled B) eluting at ∼17 ml (Fig. 1 ▸*a*).

The fractions corresponding to peaks A and B were separately pooled and concentrated for subsequent size-exclusion chromatography coupled to multi-angle light-scattering (SEC-MALS) analysis. Analysis of the SDS–PAGE and anti-His Western blot (His_6_ tag on the heavy chain of the Fab) of these samples indicated the presence of the heavy and light chains under reducing conditions and of intact Mannitou Fab under nonreducing conditions (Fig. 1 ▸*b*). SEC-MALS analysis of peak A using a d*n*/d*c* ratio of 0.185 ml g^−1^ revealed a molecular weight of 49.6 kDa for the protein (Fig. 1 ▸*c*). These results suggest that recombinantly produced Mannitou Fab predominantly exists as a canonical monomer. The application of a d*n*/d*c* ratio of 0.15 ml g^−1^ for glycoconjugation analysis indicated a glycan content of ∼3 kDa on Mannitou Fab.

The molecular weight of 101.7 kDa determined for peak B using SEC-MALS was approximately twice that of the monomeric Mannitou Fab, corresponding to a potential Fab dimer (Fig. 1 ▸*c*). SDS–PAGE, Western blot and proteomics analyses confirmed the presence of His_6_-tagged Mannitou Fab in this peak (Fig. 1 ▸*b*). However, whereas the nonreduced sample ran as expected, most of the TCEP-reduced sample migrated at a higher molecular weight. This is a phenomenom that is often observed for amyloids and may suggest that peak B contains a misfolded protein (Belashova *et al.*, 2023 ▸; Kryndushkin *et al.*, 2013 ▸). Also, it has been reported that antibodies can form amyloid structures (Wall *et al.*, 2004 ▸) and that Fab fragments can form domain-swapped dimers (Luo *et al.*, 2017 ▸; Shahid *et al.*, 2021 ▸); therefore, we wanted to explore peak B further using SEC-SAXS.

### 3.2. SAXS study of recombinant Mannitou Fab

**Figure 2:**
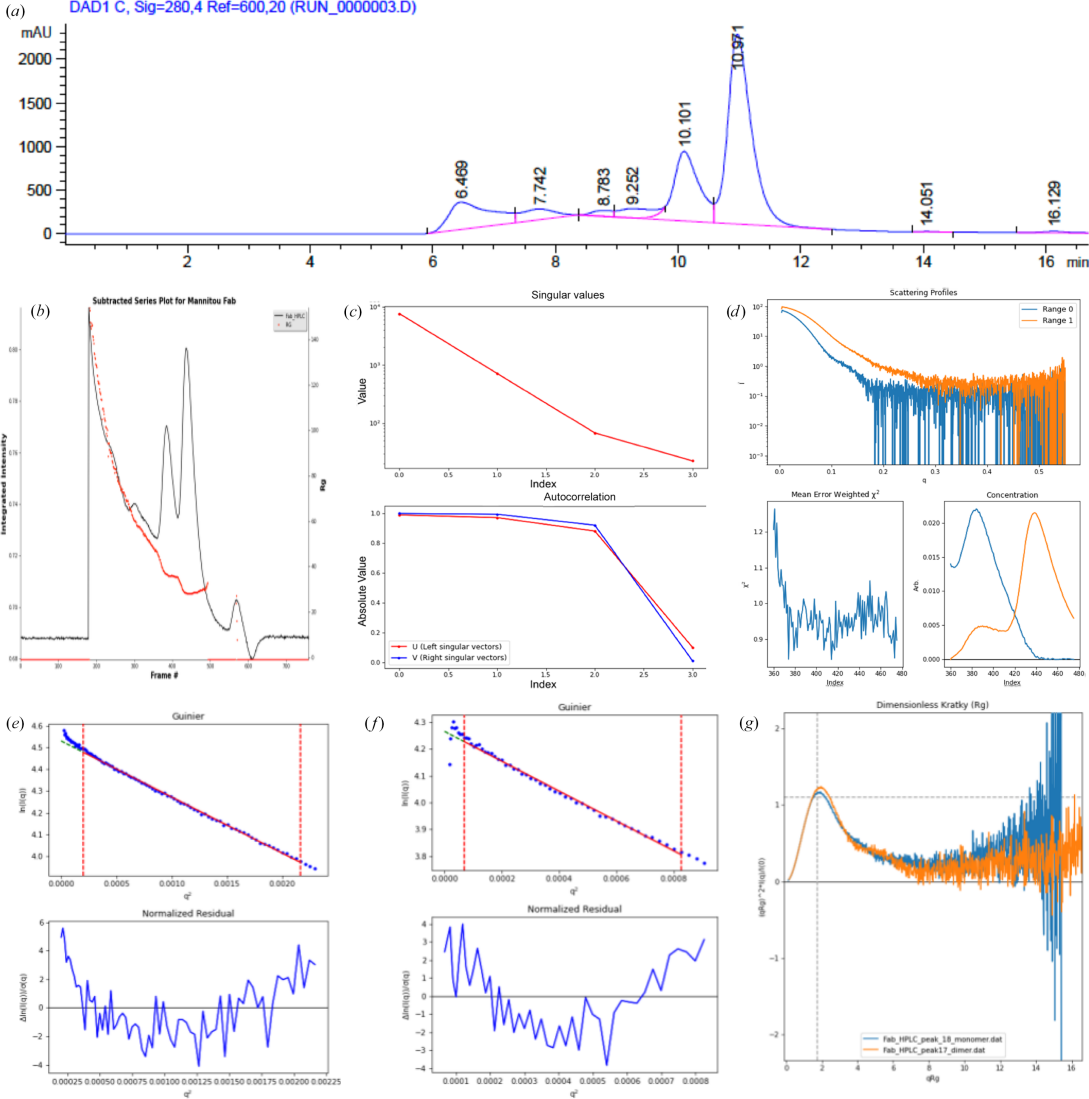
SEC-SAXS profile of the Mannitou Fab sample on a Bio SEC-3 300 column. (*a*) Absorption at 280 nm, (*b*) small-angle X-ray scattering, (*c*) singular value decomposition and (*d*) evolving factor analysis. Advanced SEC-SAXS processing used the *BioXTAS RAW* software. (*e*) Guinier plot analysis for the dimer, (*f*) Guinier plot analysis for the monomer and (*g*) a dimensionless Kratky plot indicate that both proteins are well folded.

As in SEC-MALS, the SEC-SAXS profile of the affinity-purified Mannitou Fab sample displayed two peaks eluting at 10.1 min (3.03 ml) and 10.91 min (3.27 ml) using UV detection at 280 nm (Fig. 2 ▸*a*). The small-angle X-ray scattering profile matched the SEC profile, showing two overlapping peaks in the data set (Fig. 2 ▸*b*). In *BioXTAS RAW*, singular value decomposition (SVD) and evolving factor analysis (EFA) are essential for analysing SAXS data from complex mixtures. SVD, as the first step in EFA, isolates and quantifies overlapping scattering contributions from monomeric and oligomeric species by simplifying the data and reducing noise (Fig. 2 ▸*c*). EFA builds on SVD to separate these contributions further, enabling the precise analysis of individual components, although neither method identifies the dominant species in each peak (Fig. 2 ▸*d*).

SEC-MALS and SEC-SAXS analyses revealed oligomeric states of Mannitou Fab (Figs. 1 ▸ and 2 ▸*a*). The pre-eluting peaks for Mannitou Fab from SEC (Fig. 1 ▸*a*) would agree with multimerization of the Fab monomer from peak A. Therefore, we also attempted to fit a Fab dimer to the SEC-SAXS data for the peak eluting at 10.1 min (3.03 ml) (Fig. 2 ▸). The *CRYSOL* program (Svergun *et al.*, 1995 ▸) was used to compare different monomeric and dimeric models with the data. The dominant peak at 3.27 ml was best fitted by a monomeric model (χ^2^ = 6.2), whereas a domain-swapped dimer model best fitted the secondary peak at 3.03 ml (χ^2^ = 8.6), indicating a significant dimer population in peak B and a predominantly monomeric population in peak A. This also confirmed that the two peaks from the Bio SEC-3 300 column were effectively separated using EFA in *BioXTAS RAW* (Nielsen *et al.*, 2009 ▸).

Among other structural parameters, SAXS can determine the radius of gyration (*R*_g_) and the maximum dimension (*D*_max_) of the protein (Fig. 2 ▸). Analysis of SAXS data using *BioXTAS RAW* (Nielsen *et al.*, 2009 ▸) revealed that the most intense peak eluting at 3.27 ml has a molecular mass of about ∼50 kDa, which agrees with the size of monomeric Mannitou Fab. Monomeric Mannitou Fab was thus the major product found, with a molecular mass of approximately 50 kDa, a radius of gyration (*R*_g_) of 28 Å and a maximal width (*D*_max_) of 149 Å (Table 2 ▸). The peak eluting at 3.03 ml was estimated to correspond to a molecular mass greater than ∼130 kDa and was therefore difficult to identify as a dimeric Fab (Table 2 ▸). This is likely to be the consequence of contamination with higher-order species.

### 3.3. Modelling of glycosylated Fab to fit the SAXS data

**Figure 3:**
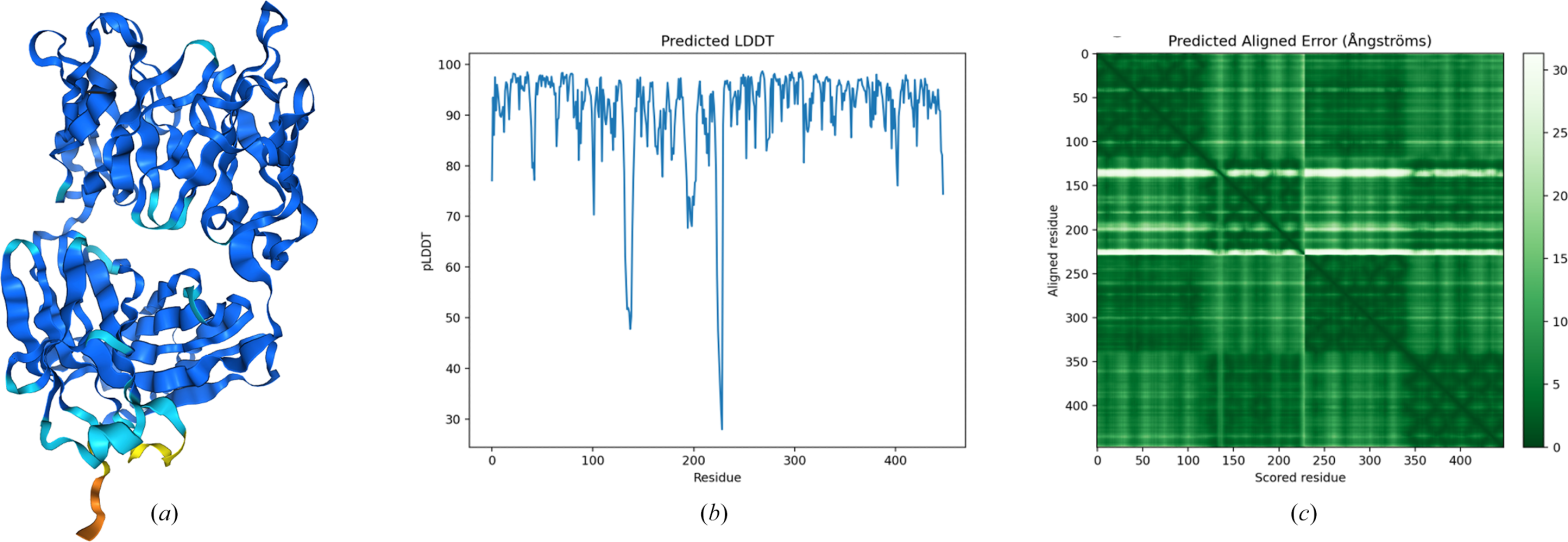
*AlphaFold*3 model of Mannitou Fab and confidence assessment. (*a*) Predicted 3D structure of Mannitou Fab using *AlphaFold*3. The model is colour-coded based on predicted local distance difference test (pLDDT) scores, where blue represents high confidence (>90), cyan indicates medium confidence (70–90) and orange/yellow highlights regions with lower confidence (<70), typically corresponding to flexible or disordered areas. (*b*) pLDDT scores plotted for each residue in Mannitou Fab (sequence as shown in Table 1 ▸) showing high confidence for most regions, with occasional dips in flexible or disordered loops. (*c*) Predicted aligned error (PAE) matrix illustrating the relative confidence in the spatial alignment of residue pairs. Dark green indicates low alignment error (high confidence), while lighter shades signify areas of greater positional uncertainty.

Small-angle X-ray scattering (SAXS), although limited in its ability to provide structural information at high resolution, provides valuable insights into the overall shape and conformational dynamics of biological macromolecules in solution. To investigate the solution structure of recombinantly engineered Mannitou Fab, possible models retrieved both from the PDB and using *AlphaFold*3 predictions (Abramson *et al.*, 2024 ▸) were fitted to the SEC-SAXS data using *CRYSOL* (Schneidman-Duhovny *et al.*, 2013 ▸) from the *ATSAS* package (Svergun *et al.*, 1995 ▸). The *AlphaFold*3 model of Mannitou Fab could be predicted with high confidence, as shown by local and global validation metrics such as pLDDT and PAE (Fig. 3 ▸). However, the fit to the data was moderate, with a lowest obtained χ^2^ value of 10.5. This discrepancy likely arose because the models lacked N-linked glycosylation, a predicted post-translational modification of Mannitou Fab.

**Figure 4:**
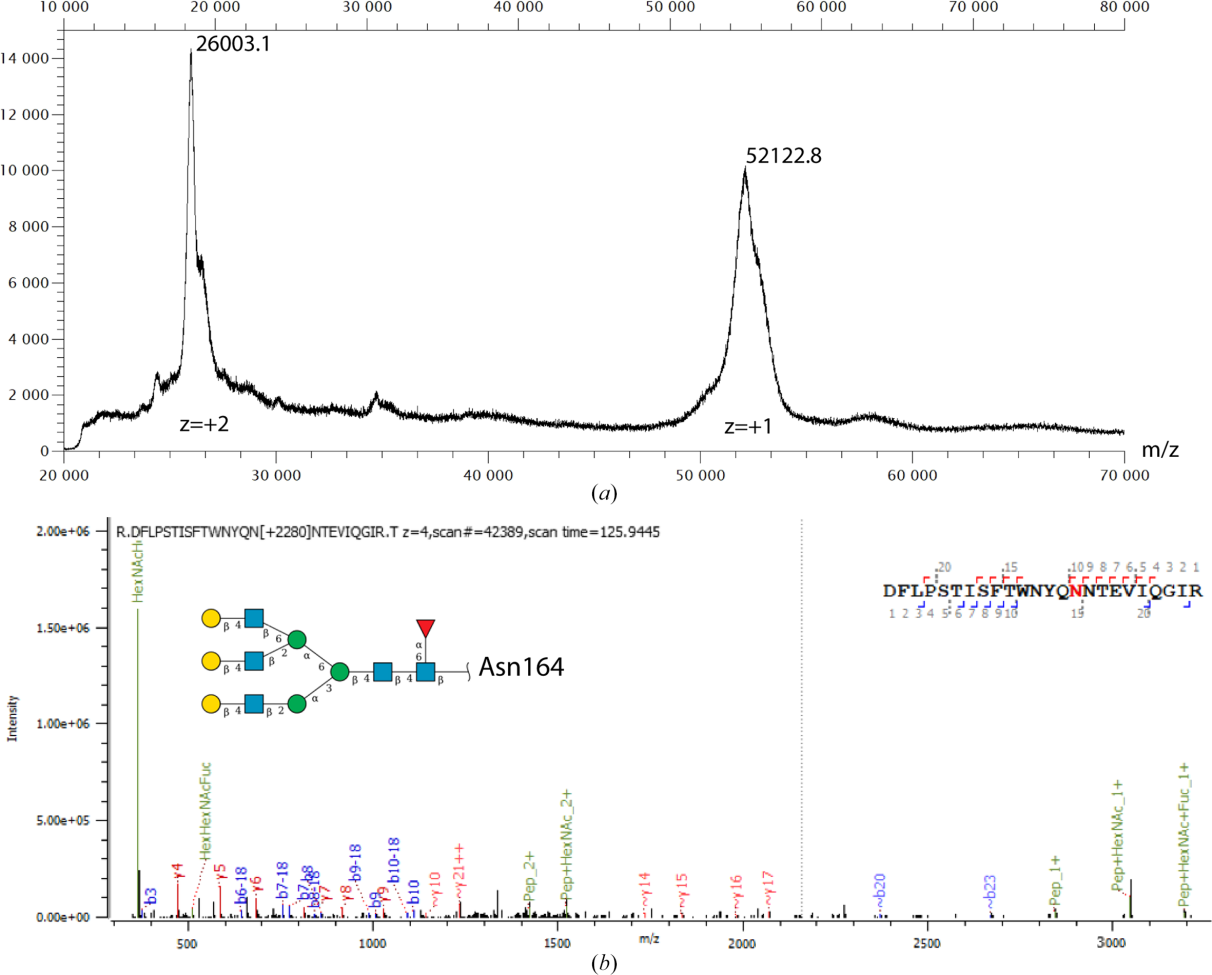
Mass-spectrometric analysis of monomeric Mannitou Fab. (*a*) Linear MALDI-TOF in positive-ion mode revealed a surplus mass of 2380 Da on Mannitou Fab. (*b*) LC-MS-MS data analysis of the glycopeptide containing Asn164 identifies the presence of a complex N-glycan including 1–3 fucoses; the glycan structure shown was used for modelling.

Sequence analysis of Mannitou Fab using the *NetNGly*1.0 server (Gupta & Brunak, 2002 ▸) identified two potential N-glycosylation consensus sequences at residues Asn58 and Asn164 in the heavy chain. Mannitou Fab, which elutes at ∼18 ml from the Superose 6 SEC column (Fig. 1 ▸*a*), has a molecular mass of 52 122.8 Da, as revealed using linear MALDI-TOF in positive-ion mode (Fig. 4 ▸*a*). This is 2380 Da above the theoretical molecular mass for the protein only (49 742.8 Da), congruent with the ∼3 kDa glycan content mass as calculated from the SEC-MALS glycoconjugate analysis, thus indicating post-translational modifications.

Bioinformatics analysis of the LC-MS-MS data using *Byonic* (Bern *et al.*, 2012 ▸) identified that Asn164, the first residue in the N-glycosylation consensus sequence NNT, carries a complex glycan (Fig. 3 ▸*b*) with the composition HexNAc(5)Hex(6)Fuc(1–3) (Fig. 4 ▸*b*). These data have been deposited in the PRoteomics IDEntifications (PRIDE; Perez-Riverol *et al.*, 2022 ▸) database under accession number PXD057808.

In the best-scoring *AlphaFold*3 model (Fig. 3 ▸), the complex N-linked glycan, HexNAc(5)Hex(6)Fuc(1) (Fig. 4 ▸*b*), was incorporated using the *GlycoSHIELD* server (Tsai *et al.*, 2024 ▸). Only one fucose, α1,6-linked to the glycan core (Fig. 4 ▸*b*), was taken into the glycan structure because of the ambiguous position of the other fucose residues on the glycan tree.

**Figure 5:**
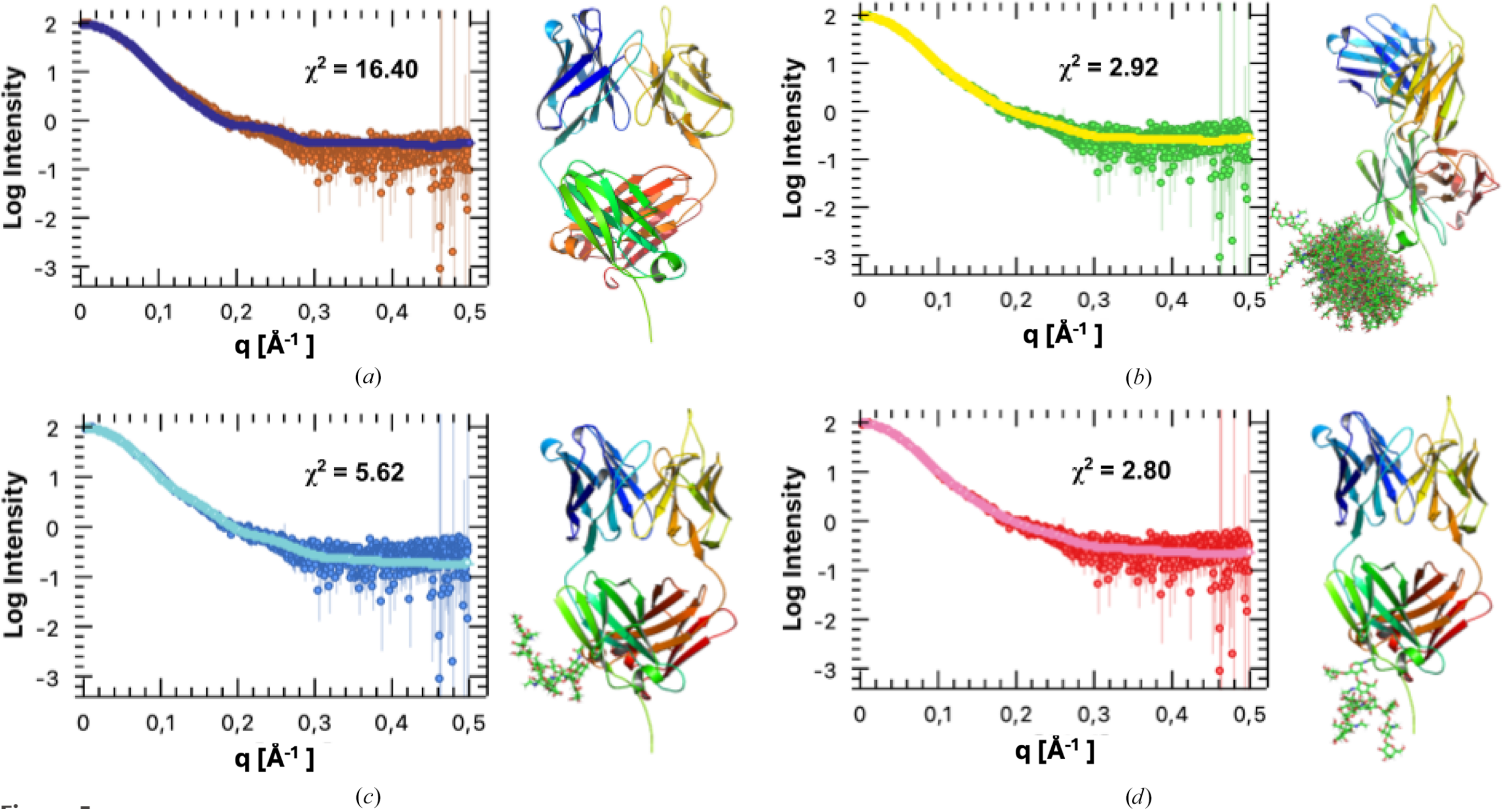
Mannitou Fab fits the SAXS data optimally upon N-linked glycan conjugation. The theoretical scattering curves of the *AlphaFold*3 models for Mannitou Fab were calculated (solid lines) and compared with the experimental SAXS data (dots); the respective models used for the calculations are shown on the right side of each fit to the SAXS curve. (*a*) Highest scoring model obtained from unmodified *AlphaFold*3 without glycosylation, (*b*) multi-state model generated using *GlycoSHIELD*, (*c*) lowest scoring glycan conformer of Mannitou Fab and (*d*) highest scoring glycan conformer of Mannitou Fab.

Using the *GlycoSHIELD* server, we generated an ensemble of 30 glycan conformations that were fitted to the SAXS data (Fig. 5 ▸*b*). The ensemble of glycosylated Mannitou Fab models showed a significant improvement (χ^2^ = 2.92) over the non­glycosylated Fab model, confirming the validity of the adjusted model. Evaluation of the 30 different glycan conformations one by one, using the *FoXS* (Schneidman-Duhovny *et al.*, 2016 ▸) server, demonstrated a χ^2^ value ranging from 5.6 (least well fitting) to 2.8 (best fitting), with the latter indicating a good fit to the SAXS data, as χ^2^ values between 1.0 and 3.0 generally reflect a good model-to-data agreement (Figs. 5 ▸*c* and 5 ▸*d*; Trewhella, 2023 ▸). *BilboMD* (Pelikan *et al.*, 2009 ▸) simulation of the glycosylated model similarly improved the fit over the rigid *AlphaFold*3 model, with a χ^2^ value of 3.1. These results underscore the critical role of glycosylation in stabilizing the Mannitou Fab monomer and achieving superior agreement with experimental SAXS data, with the χ^2^ value of 2.8 highlighting the robustness and accuracy of the glycosylated model.

### 3.4. Structural states of Mannitou Fab in solution via *MultiFoXS*

*MultiFoXS*, a computational method for interpreting SAXS data from heterogeneous samples, was employed to explore the possibility of multiple conformational states of Fab in solution (Schneidman-Duhovny *et al.*, 2016 ▸). We hypothesized that Fab might exist in multiple conformational states that could not be adequately fitted by a single model in SAXS data (χ^2^ = 2.80). We evaluated different conformational ensembles to improve the fitting scores by identifying the flexible inter-domain linkers in the unmodified *AlphaFold*3 model in *MultiFoXS*. In the absence of glycosylation of the model, the constant domain of the heavy chain dissociated from the constant domain of the light chain in one of the different conformer states. Such dissociation has been described as leading to alternative folding states (Feige *et al.*, 2010 ▸), misfolding and domain swapping (Calarese *et al.*, 2003 ▸), sometimes with the formation of dimeric Fabs, consistent with the literature on engineered Fab fragments (Luo *et al.*, 2017 ▸; Shahid *et al.*, 2021 ▸).

Yet, glycosylation of the monomeric Fab model with the complex N-linked glycan HexNAc(5)Hex(6)Fuc(1) (Fig. 4 ▸*b*) using *GlycoSHIELD* (Tsai *et al.*, 2024 ▸) prevented the fitting towards multi-state conformers. Indeed, the *MultiFoXS* analysis for the glycosylated Fab was consistent with the previous results from *FoXS* and *BilboMD* in that a single conformer of Mannitou Fab best fitted the data (χ^2^ = 2.8). This again confirms the importance of a complete Fab model inclusive of glycosylation for fitting the SAXS data.

### 3.5. The presence of dimeric Fab in the elution profile and SRCD analysis of the origin of Fab oligomerization

Attempts to fit dimeric Mannitou Fab models to SAXS profiles revealed that *AlphaFold*3 could not accurately predict dimeric Fab models. *MassiveFold* (Raouraoua *et al.*, 2024 ▸) provided additional options and also included domain-swapped dimers. A domain-swapped dimer predicted by *MassiveFold* performed better in fitting (χ^2^ = 8.6) compared with the ‘kissing’ Fab model with opposing antigen-binding sites (χ^2^ = 19.6). However, the presence of only complex glycans on the Mannitou Fab monomer does not support interaction between Fab monomers through the glycosylation as the onset of dimerization into ‘kissing’ Fabs, because of the specificity of Mannitou Fab uniquely for paucimannosidic glycans (Robakiewicz *et al.*, 2021 ▸; Zipser *et al.*, 2012 ▸). The high χ^2^ values could be due to peak B containing a mixture of dimers and higher-order species (Fig. 2 ▸*a*). Glycosylation of the dimeric models improved the fitting slightly, but the scores remained suboptimal. *BilboMD* resulted in the best fit for the dimeric Fab peak B (χ^2^ = 5.2) by giving flexibility to the linker residues between the Fab domains. However, this solution involved placing two Fabs in close proximity without sharing a dimer interface and these two Fabs would be expected to elute together with the Mannitou Fab monomer in peak A of the SEC column. Instead, the Mannitou Fab dimer population in peak B is possibly attributable to other factors, such as domain swapping (Calarese *et al.*, 2003 ▸), which sometimes results in the the formation of dimeric Fabs, consistent with the literature on engineered Fab fragments (Luo *et al.*, 2017 ▸; Shahid *et al.*, 2021 ▸).

**Figure 6:**
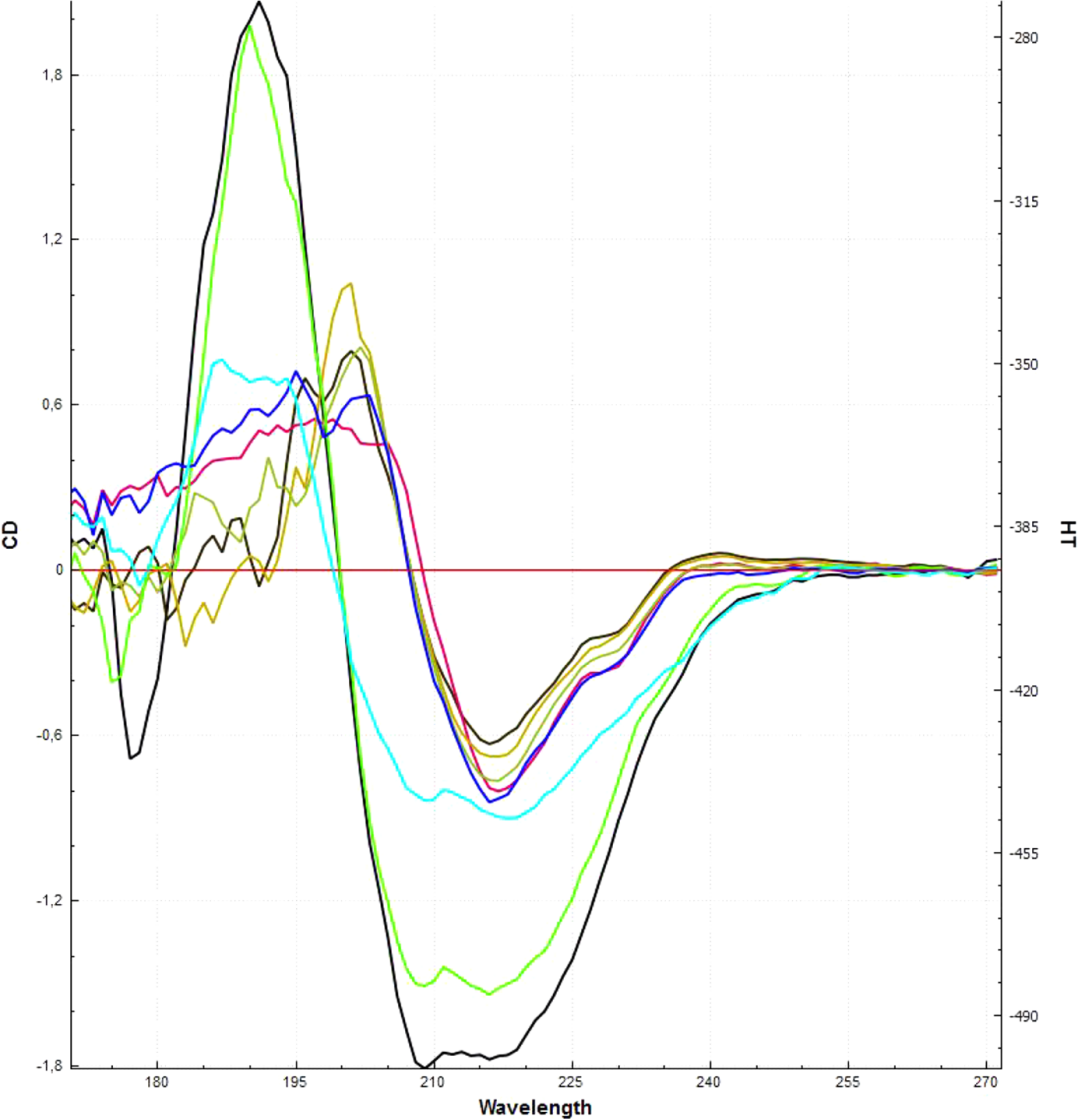
Overlay of SRCD spectra of Mannitou Fab conformations. Five ‘peak A’ Fab samples (dark brown to dark blue) and two ‘peak B’ Fab samples (light green and black) are shown, as well as the monomer Fab from peak A transitioned from 20 to 80°C in only 3 min (cyan). There is a shift to the left with a loss of antiparallel β-sheet structure.

For these reasons, we undertook SRCD on Mannitou Fab peaks A and B (Fig. 1 ▸) to also evaluate their secondary-structure content using *BeStSEL* (Micsonai *et al.*, 2021 ▸). The Mannitou Fab peak A sample was also heated from 20°C to 80°C in just 3 min to evaluate whether it undergoes structural alterations. This appeared to indeed be the case when comparing the spectra (Fig. 6 ▸). SRCD spectra indicate that the structure of Mannitou Fab in peak A differs from that in peak B despite their similar band pattern on the denaturing gel (Fig. 1 ▸*b*). A significant difference in secondary structure in the first eluting peak from the SEC column, peak B, is apparent, with a reduction of antiparallel β-sheet from more than 50% for monomeric Mannitou Fab in peak A to ∼20% in peak B. Upon heat denaturation, the Mannitou Fab monomer undergoes a similar transition, with a reduction in antiparallel β-sheet to 26% (Fig. 6 ▸, cyan). The altered conformation of Fab could be due to misfolding with altered interdomain disulfide formation (Gani *et al.*, 2020 ▸) or other types of misfolding with structural rearrangements (Feige *et al.*, 2010 ▸), deviating from the best-case scenario as found in some crystallized Fabs where domains associate with a domain from another Fab assembly (swapped) while maintaining (Shahid *et al.*, 2021 ▸) or even enhancing (Calarese *et al.*, 2003 ▸) antigen binding. Consequently, we did not have a model to fit into the SAXS data or peak B, as no dimeric Fab model is credible.

## 4. Conclusions

The successful isolation and concentration of Mannitou Fab was validated using SEC-MALS and SEC-SAXS. The prominent peak eluting from SEC columns corresponds to the molecular mass of monomeric Fab and could also be validated as such. A series of earlier eluting peaks suggest that oligomeric species, including Fab dimers, are also present to a lesser extent. These results indicate that while the monomeric form is the predominant and most stable form, Mannitou Fab may undergo aggregation in the form of higher-order structures. This is potentially due to the relatively long expression time in the culture medium, freezing and thawing cycles, concentration of the protein and other destabilizing factors. The homogeneity of protein samples is becoming even more important in the context of the increasing application of electron microscopy for the structural evaluation of antibodies.

The improved fit of the monomeric model in SAXS analysis, after accounting for glycosylation and structural states in solution, underscores the importance of incorporating these factors into the design process to ensure structural stability and functional efficacy. Moreover, the challenges encountered in modelling the dimeric forms of Fab, particularly the discrepancies in molecular mass and flexibility, emphasize the need for further refinement of computational tools. Current modelling approaches, including *AlphaFold*3 and *MassiveFold*, demonstrate potential but also highlight limitations in predicting complex antibody structures, especially in the presence of post-translational modifications such as glycosylation, and in predicting misfolding of multi-domain proteins. Future developments in computational modelling could focus on better integrating data on post-translational modifications and improving the accuracy of models for the multi-domain antibody structures.

This study provides a biochemical and biophysical analysis of Mannitou Fab, highlighting the intricate balance between stability and function in engineered antibody fragments. To build on the insights gained from this study, research could aim to explore the functional implications of glycosylation in Fab fragments, particularly its role in modulating interactions with antigens and other biomolecules. For example, given the affinity of Mannitou for paucimannose-carrying glycoproteins (Robakiewicz *et al.*, 2021 ▸), it was essential in our SAXS study of Mannitou Fab to decipher the glycosylation and exclude the possibility that oligomeric populations arose from intermolecular interactions between Fabs via glycosylation at these sites. Additionally, integrating SAXS with complementary techniques such as cryo-electron microscopy (cryo-EM) and nuclear magnetic resonance (NMR) spectroscopy could provide a more comprehensive view of the structural dynamics of Fab molecules in order to overcome some of the limitations observed in SAXS-based modelling and to pave the way for more effective and reliable therapeutic antibodies.

## Supplementary Material

SASBDB reference: Mannitou Fab, SASDWK2

## Figures and Tables

**Table 1:** Mannitou Fab production information

Source organism	*Mus musculus*
DNA source	Synthetic, GenScript
Expression vector	pHLSec
Expression host	HEK293 FreeStyle cells
Molecular weight (Da)	49742.8
Complete amino-acid sequence	
Heavy chain VH-Cμ1-His_6_	EVKLLESGGGLVQPGGSLKLSCAASGFDFSTYWMSWVRQAPGKGLEWIGEINPDSSTINYTPSLKDKFIISRDNAKNTLYLQMSKVRSEDSVLYYCVRPGTWGYFDYWGQGTTLTVSSESQSFPNVFPLVSCESPLSDKNLVAMGCLARDFLPSTISFTWNYQNNTEVIQGIRTFPTLRTGGKYLATSQVLLSPKSILEGSDEYLVCKIHYGGKNRDLHVPIPHHHHHH
Light chain VL-CL	DVVVTQTPLSLPVSFGDQASISCRSSQSLVNSYGSTYLSWYLHRPGQSPQLLIYGISNRFSGVPDRFSGSGSGTDFTLTIRTIKPEDLGMYYCLQGTHQPWTFGGGTKLEIKRADAAPTVSIFPPSSEQLTSGGASVVCFLNNFYPKDINVKWKIDGSERQNGVLNSWTDQDSKDSTYSMSSTLTLTKDEYERHNSYTCEATHKTSTSPIVKSFNRKEC

**Table 2:** SEC-SAXS data collection, processing and model refinement *I*(0), extrapolated scattering intensity at zero angle; *R*_g_, radius of gyration calculated using Guinier approximation; MM, molecular mass; *D*_max_, maximal particle dimension; *V*_p_, Porod volume.

	Fab peak 3.03 ml	Fab peak 3.27 ml
Data-collection parameters		
Beamline	SWING, SOLEIL	SWING, SOLEIL
Wavelength (Å)	0.99	0.99
*q* range (Å^−1^)	0.004–0.551	0.004–0.551
Concentration (mg ml^−1^) (mode)	9.2 (SEC-SAXS)	9.2 (SEC-SAXS)
Buffer conditions	20 m*M* HEPES, 300 m*M* NaCl pH 7.5	20 m*M* HEPES, 300 m*M* NaCl pH 7.5
Temperature (°C)	15	15
Structural parameters		
*I*(0) (from Guinier) (cm^−1^)	70.93	93.22
*R*_g_ (from Guinier) (Å)	40.35	28.03
*D*_max_ (Å)	∼216	∼149
Porod volume estimate, *V*_p_ (Å^3^)	175299	64600
Molecular-mass determination		
MM (from *SAXSMoW* on final merged curve) (kDa)	150	53.1
MM (from size and shape) (kDa)	152.9	50.9
MM (from Bayesian inference) (kDa)	130.9	47.7
Calculated MM from sequence (kDa)	49.7	49.7
Ensemble modelling		
Conformer generation and selection	*BilboMD*	*BilboMD*
Residues selected to be fixed/rigid	1–118, 1–112/123–221, 115–216	1–118, 1–112/123–221, 115–216
Software employed		
Data processing and analysis	*ATSAS*, *RAW*	*ATSAS*, *RAW*
Computation of theoretical intensities and fitting	*FoXS*, *MultiFoXS*	*FoXS*, *MultiFoXS*
Flexible residues for fitting	118–123, 112–115	118–123, 112–115
SASBDB code		SASDWK2
